# Experimental Investigation of Actively Q-Switched Er^3+^:ZBLAN Fiber Laser Operating at around 2.8 µm

**DOI:** 10.3390/s20164642

**Published:** 2020-08-18

**Authors:** Lukasz Sojka, Lukasz Pajewski, Samir Lamrini, Mark Farries, Trevor M. Benson, Angela B. Seddon, Slawomir Sujecki

**Affiliations:** 1 Department of Telecommunications and Teleinformatics, Faculty of Electronics, Wroclaw University of Science and Technology, Wybrzeze Wyspianskiego 27, 50-370 Wroclaw, Poland; lukasz.pajewski@pwr.edu.pl (L.P.); slawomir.sujecki@pwr.edu.pl (S.S.); 2 LISA Laser Products GmbH, Albert-Einstein-Straße 4, 37191 Katlenburg-Lindau, Germany; slamrini@lisalaser.de; 3 Gooch & Housego Ltd., Dowlish Ford, Ilminster, Somerset TA19 OPF, UK; Mark.Farries@nottingham.ac.uk; 4 Mid-Infrared Photonics Group, George Green Institute for Electromagnetics Research, Faculty of Engineering, University of Nottingham, University Park, Nottingham NG7 2RD, UK; Trevor.Benson@nottingham.ac.uk (T.M.B.); Angela.Seddon@nottingham.ac.uk (A.B.S.)

**Keywords:** mid-infrared light sources, fluoride glass fibers, mid-infrared, fiber lasers

## Abstract

A diode-pumped Q-switched Er^3+^:ZBLAN double-clad, single-transverse mode fiber laser is practically realized. The Q-switched laser characteristics as a function of pump power, repetition rate, and fiber length are experimentally investigated. The results obtained show that the Q-switched operation with 46 µJ pulse energy, 56 ns long pulses, and 0.821 kW peak power is achieved at a pulse repetition rate of 10 kHz. To the best of our knowledge, this is the highest-ever demonstrated peak power emitted from an actively Q-switched, single-transverse mode Er^3+^:ZBLAN fiber laser operating near 2.8 µm.

## 1. Introduction

Mid-infrared (MIR) fiber lasers with emitting wavelength near to 3 µm have numerous applications in sensing [1,2,3,4]. This is because these MIR fiber lasers exploit one of the most informative regions of the electromagnetic spectrum for molecular recognition. For instance, a very convenient way of accurately measuring the concentration of atmospheric gasses consists of the application of mid-infrared lidars. MIR lidars are especially useful when operating at low altitudes and in marine environments [5]. This is because lidars operating in the MIR region are less sensitive to scattering, turbulence, and humidity [5]. However, in order to realize a mid-infrared lidar the development of a laser with the following features is necessary: high output energy, short pulse duration (to reduce the impact of thermal background), good pulse-to-pulse stability, and diffraction limited beam quality. The laser should also be compact and robust [5,6]. All these requirements predestine a pulsed mid-infrared fiber laser as a suitable light source for lidar systems. This point has been recently demonstrated experimentally by a research team from Australia [4]. In reference [4] a swept-wavelength (2.8–3.4 μm) Dy^3+^:ZBLAN (composed of fluorides: ZrF_4_, BaF_2_, LaF_3_, AlF_3_, and NaF) fiber laser was used for the real time sensing of ammonia gas. Therefore, the development of a mid-infrared fiber laser for mid-infrared gas sensors is the main subject of this contribution, since such a device provides the key enabling technology.

Moreover, water is one of molecules that poses a set of strong absorption lines in the MIR region near 3 µm. Thus, MIR fiber lasers can find applications in laser surgery, because human tissue contains a large proportion of water [7]. Additionally, fiber lasers emitting at around 3 µm can be used for micromachining polymer materials that have strong MIR absorption [8]. Lately, Churbanov et al. demonstrated the first 5 µm laser action in a terbium (III) doped selenide glass rod. Interestingly, in order to obtain 5 µm lasing, the terbium (III) doped selenide glass rod was pumped by using a pulsed Er^3+^:YAG (yttrium aluminium garnet) solid-state laser operating at 2.9 µm [9]. Thus, in the future, to generate 5 µm laser action in terbium (III) doped selenide fibers pumping with a ~3 µm pulsed fiber laser should be preferred. Moreover, a single-transverse mode pulsed fiber laser source with high peak power facilitates supercontinuum generation in small-core nonlinear fibers [10,11].

Currently, in the 3 µm wavelength region, two fiber laser types can achieve short pulse operations in the range of ns and pulse energies up to 10s µJ, i.e., active Q-switched and gain-switched (GS) lasers [12,13]. Furthermore, Er^3+^:ZBLAN femtosecond mode-locking laser using a nonlinear polarization rotation mechanism has also been reported [14]. However, the pulse energies achieved in reference [14] were less than 10 nJ. In reference [12], Tokita et al., 2011 presented a multi-transverse mode actively Q-switched Er^3+^-doped ZBLAN fiber laser whereby Q-switching was implemented using an acousto-optic modulator (AOM). In reference [12], the realized laser was producing pulses as short as 90 ns at a repetition rate of 120 kHz with peak powers of 0.9 kW. More recently, Shen et al. presented a multi-transverse mode Q–switched Er^3+^:ZBLAN fiber laser with an average power of 1.5 W and a peak power up to 1.6 kW (pulse energy of 0.15 mJ) at 10 kHz, realizing the Q-switching by using mechanical modulator placed within the laser cavity [15]. The maximum peak power achieved so far from a multi-transverse mode Q-switched fluoride glass fiber laser is larger than 10 kW [16]. Additionally, single-transverse mode gain-switched Er^3+^:ZBLAN all-fiber lasers have been demonstrated [13]. In reference [13], the realized laser had a maximum average output power of 11.2 W, pulse energy of 80 μJ, and pulse duration 170 ns at the operating wavelength of 2.826 µm. Recently, also, Q-switched and gain-switched Dy^3+^: ZBLAN fiber lasers operating were reported [17,18]. Furthermore, in reference [19], the first actively Q-switched fiber laser operating near a wavelength of 3.5 μm was reported. In reference [19], robust Q-switching and a pulse energy of 7.8 μJ at a repetition rate of 15 kHz, corresponding to a peak power of 14.5 W, was achieved. The authors also pointed out in this contribution an interesting problem with damage of the fiber tip observed for higher pump powers. Moreover, a large effort has been invested in the numerical modeling of Q-switched and gain-switched (GS) 3 µm fiber lasers [17,20,21,22,23]. In 2012, Hu et al. reported a single-transverse mode Q-switched Ho^3+^, Pr^3+^-doped fluoride fiber laser, producing a peak power of 77 W, with a pulse width of 78 ns at a repetition rate of 120 kHz operating at 2.9 µm [24]. Further, Hu et al. demonstrated an improved single-transverse mode Q-switched Ho^3+^ and Pr^3+^-doped fluoride fiber laser operating in the first-order diffraction mode and producing pulses with a maximum peak power of 576 W and a pulse width of 33 ns at the repetition rate of 1 kHz [25]. Up to now, this was the maximum peak power obtained from a single-transverse mode actively Q-switched fluoride fiber laser operating at around 3 µm.

In this paper, we report, to the best of our knowledge, the highest ever demonstrated peak power emitted from an actively Q-switched, single-transverse mode Er^3+^:ZBLAN fiber laser operating around 2.8 µm.

The paper is divided into five sections. After this introduction, the experimental setup is explained in detail in the second section. In the third section, the experimental results are presented and discussed. In Section 4, a discussion of the results impact is provided. The last section provides a summary.

## 2. Experimental Setup

**Figure 1:**
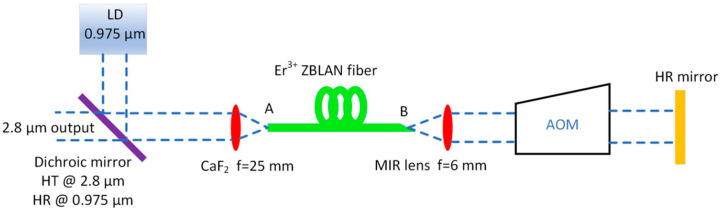
Schematic diagram of the Q-switched mid-infrared fiber laser (HR—highly reflective, HT—highly transmissive, LD—laser diode, AOM—acousto-optic modulator, and CaF_2_—calcium fluoride lens).

The experimental setup of the Q-switched Er^3+^:ZBLAN fiber laser is shown in Figure 1. The double-clad Er^3+^:ZBLAN single-transverse mode fiber was used as the gain medium. The Er^3+^:ZBLAN fiber lasers can be pumped with low-cost and high-power laser diodes at 975 nm and, also, can produce around 20–35% efficiency in the mid-infrared spectral range. A MIR emission at around 2.8 µm is expected from the ^4^I_11/2_ → ^4^I_13/2_ transition under excitation at 975 nm. Moreover, the upper-laser transition ^4^I_11/2_ has a long lifetime of 6.9 ms, which suggests the potential for gain storage [26,27]. The Le Verre Fluoré fiber used in this experiment had a 7 mol.% core doping concentration of ErF_3_, a core diameter of 15 µm, and a cladding diameter of 260 µm (NAcore = 0.126 (between fiber core and first clad) and NAclad = 0.46 (between first clad and second polymer clad) (NA is numerical aperture), with a pump absorption of approximately 3 dB/m at 0.975 µm [27]. The cladding glass of the fiber used had a double D-shape geometry, which helped to improve pump absorption, and it was coated with a low-index fluoroacrylate to enable multimode pump light guidance. The fiber was in single mode for wavelengths above 2.5 µm. In the experiment, three different Er^3+^:ZBLAN fiber lengths (1.1 m, 2.1 m, and 3.1 m) were used. The fiber ends A and B were placed in stainless-steel V-grooves, whilst the remaining part of the Er^3+^:ZBLAN fiber was attached to an aluminum plate using a thermal conductive silicone gel pad in order to improve the heat dissipation. Around 2 mm of input, the fiber end was protruding from a V-groove and was flushed by pressurized dry air in order to minimize moisture diffusion into the fiber tip. This approach also provides convection cooling of the fiber tip.

The Er^3+^:ZBLAN fiber was pumped by a 30 W maximum output power 0.975 µm multimode diode laser coupled to 105 µm multimode fiber (BWT K976DA3RN-30.00W, Beijing, China). The collimated light from the 0.975 µm pump laser was reflected by a dichroic mirror (highly reflective for wavelengths around 0.975 µm and highly transmissive for wavelengths between 2.4–3.0 µm (Layertec 109821, Mellingen, German). Then, the pump light was focused onto the fiber end facet using a plano-convex CaF_2_ lens, with a focal length of 25 mm (Eksma Optics, Vilnius, Lithuania). The fiber end A was perpendicularly cleaved and acted as an output coupler, whilst fiber end B (Figure 1) was angle cleaved at an angle of approximately 10 degrees in order to minimize the level of back reflection. The light emitted by the fiber end B was collimated using a MIR Sapphire ball lens with focal length of f = 6 mm. An acousto-optic Q-switch element was inserted into the laser cavity. The cavity was closed by a flat gold-coated high-reflector mirror with reflectance >96% (Thorlabs PF100-03-M01, Newton, MA, USA). The output power was measured using a thermal power sensor (S415C Thorlabs, Newton, MA, USA). In order to remove the pump wavelength, an optical filter with a cut-on wavelength of 2.4 µm (Edmund Optics #68-659, Barrington, IL, USA) was placed before the power meter. The measured output powers were corrected to account for the losses of the 2.4 µm cut-on filter and uncoated CaF_2_ lens. The MIR pulses were monitored using an MCT (mercury cadmium telluride) (PVMI-8 Vigo System, Ozarow Mazowiecki, Poland) photodetector (rise time < 8.8 ns) and 500 MHz bandwidth oscilloscope (LeCroy WaveSurfer 452, Heidelberg, Germany). The output spectrum generated by the fiber laser was measured using a 150 mm optical monochromator (MSH-150 LOT-Quantum Design GmbH, Darmstadt, Germany), with a diffraction grating blazed at 4 µm (MSG-S-150-4000, Ozarow Mazowiecki, Poland) and coupled to a high-sensitive thermo-electrically cooled MCT detector (Vigo System PVI-4TE-5, Ozarow Mazowiecki, Poland).

In order to obtain modulation of the laser cavity finesse, an acousto-optic Q-switch (I-QS041-1.5C2P-4-MN4 Gooch & Housego, Ilminster, UK) modulator TeO_2_ (tellurium dioxide) was used. The active Q-switch modulator had the following parameters: transmission >95% for 3 µm, active aperture 1.5 mm, loss modulation >80%, rise-time 153 ns/mm, and operating frequency of 40.68 MHz. The acousto-optic modulator (AOM) was polarization-insensitive. The active Q-switch was operating in a zero-order diffraction mode inside the laser cavity. The active Q-switch modulator was powered by a RF (radio frequency) drive (MQC041-20DC-A05-15V Gooch & Housego, Ilminster, UK) and controlled by a TTL (transistor-transistor logic) signal produced using a function generator (GW INSTEK GFG-3015, Xinbei, Taiwan). The AOM was placed on a high-precision rotational stage (Thorlabs PR01/M, Newton, MA, USA) in order to accurately adjust the Bragg angle.

## 3. Experimental Results and Discussion

### 3.1. CW (Continuous Wave) Operation of Er^3+^:ZBLAN Fiber Laser

**Figure 2:**
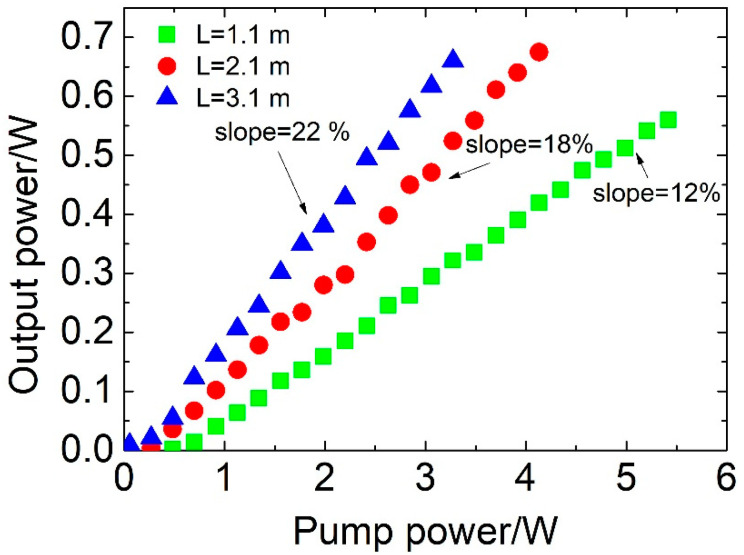
Output power of an Er^3+^:ZBLAN fiber laser as a function of the pump power for different fiber lengths. The AOM was switched off (transmission >95%).

Figure 2 shows the output power of an Er^3+^:ZBLAN fiber laser as a function of the pump power for three different fiber lengths measured with AOM in the laser cavity. The AOM was switched off (transmission >95%) so that the CW laser action was allowed. In a Q-switched fiber laser, the pulse duration depends on the pulse round-trip time [23]. By reducing the fiber length, one reduces the pulse round-trip time. For this reason, in the experiments, three different Er^3+^:ZBLAN fiber lengths (1.1 m, 2.1 m, and 3.1 m) were tested as a gain medium. The highest slope efficiency of 22% was achieved for 3.1 m of active fiber, which provided around 90% of the pump absorption (pump absorption was approximately 3 dB/m at 0.975 µm for the Er^3+^:ZBLAN fiber). Additionally, a low laser threshold of around 200 mW was measured for this laser configuration. For 2.1 m of active fiber, the slope efficiency decreased to 18%, because the pump absorption was reduced to 70%. A lower, but still reasonable, slope efficiency of 12% was recorded for 1.1 m of active fiber, which corresponded to only 50% of the pump absorption. Moreover, for this configuration, the laser threshold increased to the level of around 500 mW. The experimental results show also that the losses introduced by the AOM are very low, because the measured laser slope efficiencies with and without the AOM placed in the cavity were similar.

### 3.2. Q-Switched Operation of Er^3+^:ZBLAN Fiber Laser Containing 3.1 m of Active Fiber

**Figure 3:**
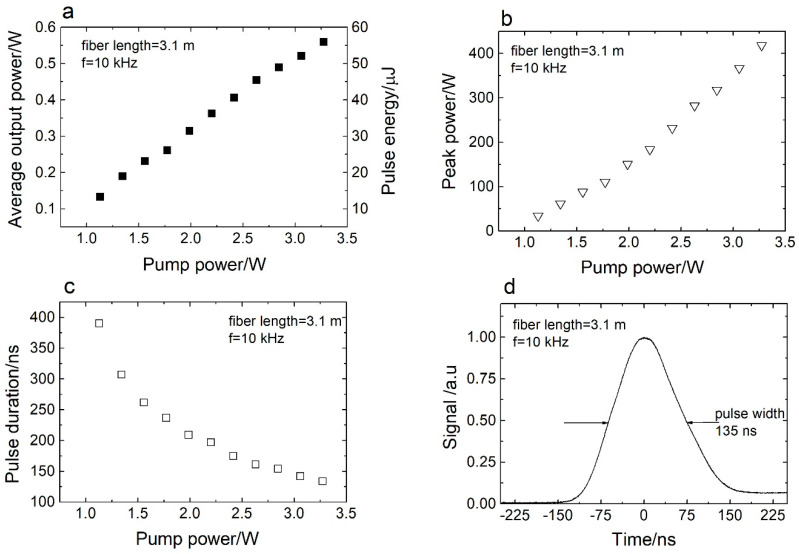
(**a**) Average output power and pulse energy versus pump power. (**b**) Peak power versus pump power. (**c**) Pulse duration versus pump power. (**d**) Pulse shape recorded at 10 kHz. The fiber length used in the experiment was 3.1 m.

The laser performance in the Q-switch regime was characterized at a repetition rate of 10 kHz. Figure 3a shows the average output power and pulse energy of the Q-switched Er^3+^:ZBLAN fiber laser as a function of the pump power. The fiber length is 3.1 m. The Q-switched laser had a slope efficiency of 20%, which was only slightly lower than in the CW regime (22%, Figure 2). The highest pulse energy obtained was 56 µJ. Further increasing the incident pump power resulted in damage to the input end of the fiber. This result is consistent with results previously published by Eichhorn [28,29], who observed the destruction of ZBLAN fiber at 53 µJ pulse energy for a 15 µm core diameter fiber. Therefore, in further experiments, the pulse energy was limited to 50 µJ. Figure 3b presents the peak power as a function of the pump power. The maximum observed peak power was 415 W for a 3.1 m long gain fiber. Figure 3c shows the pulse duration measured at the full half of the maximum for a laser oscillator consisting of 3.1 m of the Er^3+^:ZBLAN fiber as a function of the pump power. The pulse width decreases as the pump power increases. This is typical behavior for a Q-switched laser [30]. The pulse width mainly depends on the cavity round-trip time. However, it also depends on the degree of inversion [30]. Thus, by increasing the pump power, the degree of inversion is also increased, and pulses become narrower. The shortest pulse shape is shown in Figure 3d; the measured pulse duration here is 135 ns, which corresponds to four round trips.

### 3.3. Q-Switched Operation of Er^3+^:ZBLAN Fiber Laser Containing 2.1 m Active Fiber

**Figure 4:**
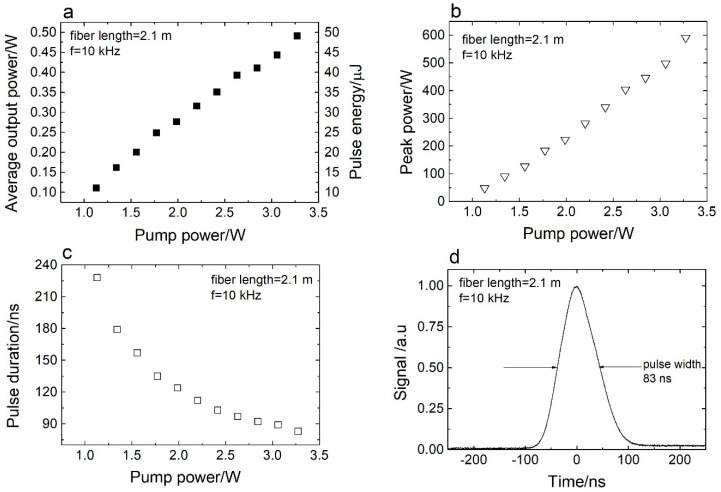
(**a**) Average output power and pulse energy versus pump power. (**b**) Peak power versus pump power. (**c**) Pulse duration versus pump power. (**d**) Pulse shape recorded at 10 kHz. The fiber length used in the experiment was 2.1 m.

Figure 4a shows the measured dependence of the average output power and pulse energy on the pump power. The fiber length used in the experiment was 2.1 m. The measured Q-switched laser slope efficiency was 17%. The maximum measured output pulse energy was 49 µJ. Figure 4b shows the peak power dependence on the pump power. It can be observed that, when the pump power increases, the output peak power also steadily increases. The maximum observed peak power was 590 W. This peak power was higher than that recorded for the cavity using 3.1 m of gain fiber. Additionally, by shortening the fiber length, the pulse round-trip time reduces. This, in turn, results in a shorter pulse duration, which is accompanied by a higher value of the peak power [23]. The evolution of the pulse duration with the pump power is presented in Figure 4c. The shortest pulse shape recorded for a cavity containing 2.1 m of gain fiber is displayed in Figure 4d. In this case, the pulse width measured at half of the maximum was 83 ns.

### 3.4. Q-Switched Operation of Er^3+^:ZBLAN Fiber Laser Containing 1.1 m of Active Fiber

**Figure 5:**
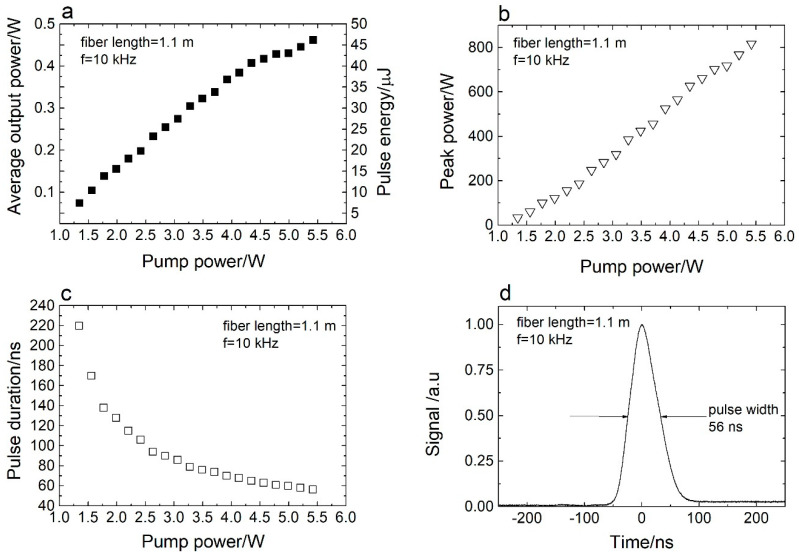
(**a**) Average output power and pulse energy versus pump power. (**b**) Peak power versus pump power. (**c**) Pulse duration versus pump power. (**d**) Pulse shape recorded at 10 kHz. The fiber length used in the experiment was 1.1 m.

The laser performance was also invesitigated for a 1.1 m length of Er^3+^:ZBLAN gain fiber. Figure 5a presents the dependence of the avarage output power and pulse energy on the 0.975 µm pump power. In this case, more pump power was needed to achieve 0.46 W average output power, due to the lower value of the pump absorption. The slope efficiency was 10% for this Q-switched fiber laser using 1.1 m of Er^3+^:ZBLAN gain fiber. In this case, only around 50% of the pump power was absorbed. Figure 5b shows the peak power as a function of the pump power. For this case, the maximum peak power increased to 821 W. This rise in the peak power value is a direct result of reducing the pulse round-trip time by reducing the fiber length. The dependence of the pulse width on the pump power is presented in Figure 5c. Figure 5d shows the shortest pulse shape. In this case, the pulse duration is 56 ns and corresponds to five to six round trips. To the best of the authors’ knowledge, this is the highest peak power obtained from an actively Q-switched ~3 µm fiber laser operating in a single-tranverse mode regime. The experimental results show that further reduction of the fiber length does not increase the peak power. This is evident, since reducing the fiber length below 1.1 m results in a lower experimentally observed average power, whilst the pulse duration stays unchanged.

**Figure 6:**
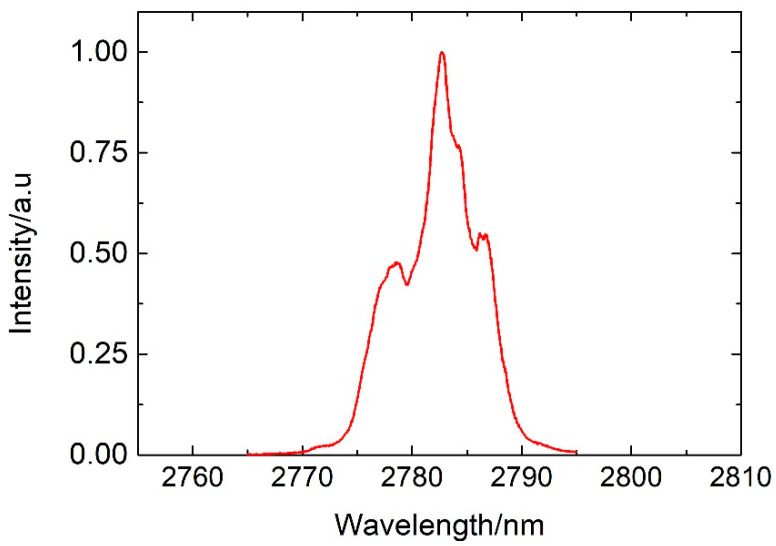
Typical Q-switched laser spectrum measured at a repetition rate of 10 kHz and pump power 3.27 W. The fiber length used in this experiment was L = 1.1 m.

Figure 6 presents the emission spectrum generated by the Q-switched laser for a pump power of 3.27 W and a 1.1 m fiber length. These results show that the laser operated at 2.783 μm with a full width at a half-maximum (FWHM) bandwidth of 7 nm.

**Figure 7:**
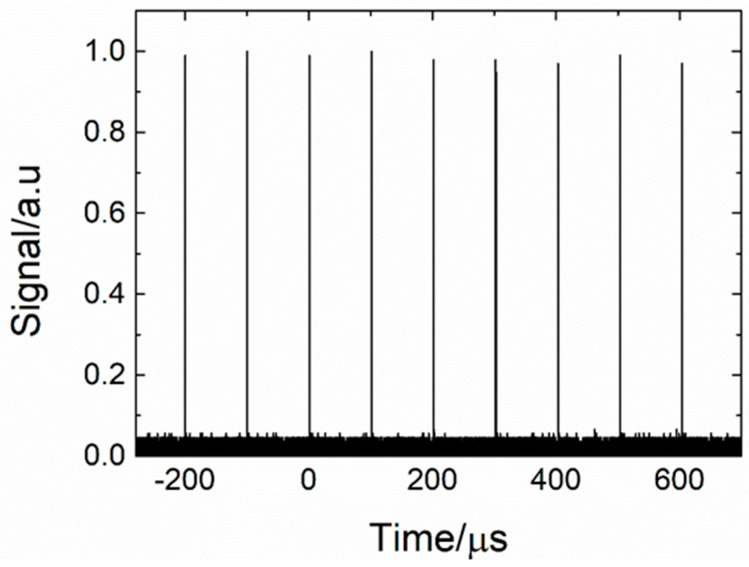
Typical 2.78 µm pulse train of a repetition rate of 10 kHz measured for a launched pump power of 3.27 W. The fiber length used in this experiment was L = 1.1 m.

Figure 7 presents examples of pulse trains recorded at the repetition rate of 10 kHz. The results were measured for a 1.1 m fiber length and a pump power of 3.27 W. The measured pulse amplitude fluctuations were below 2.0%. These results confirm that the realized laser operated in a stable manner.

**Figure 8:**
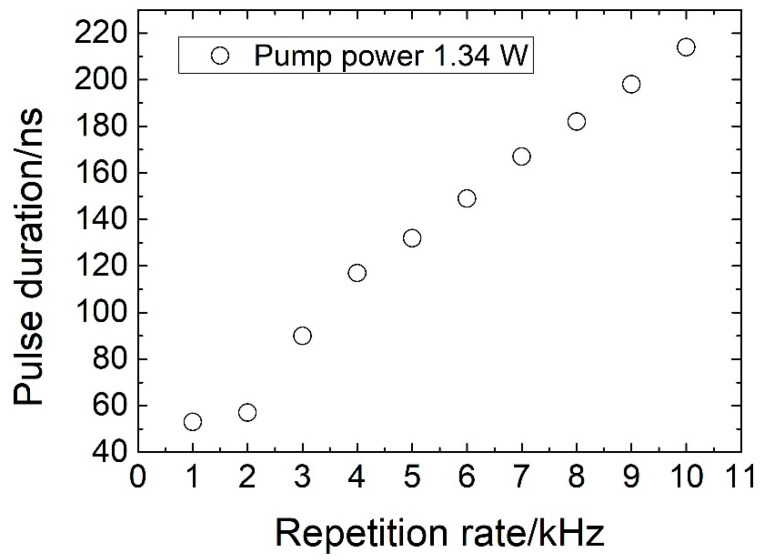
Evolution of the pulse duration as a function of the repetition rate for a launched pump power of 1.34 W. The fiber length used in this experiment was L = 1.1 m.

The influence of the repetition rate on the pulse width was also investigated (see Figure 8). The results obtained were recorded for a pump power of 1.34 W and a fiber length of 1.1 m. The results presented in Figure 8 show that the pulse duration increases with the repetition rate. The shortest pulses were produced at the lowest possible repetition rates, which is typical Q-switched laser behavior whereby a larger amount of energy stored results in shorter pulses [24,30].

**Figure 9:**
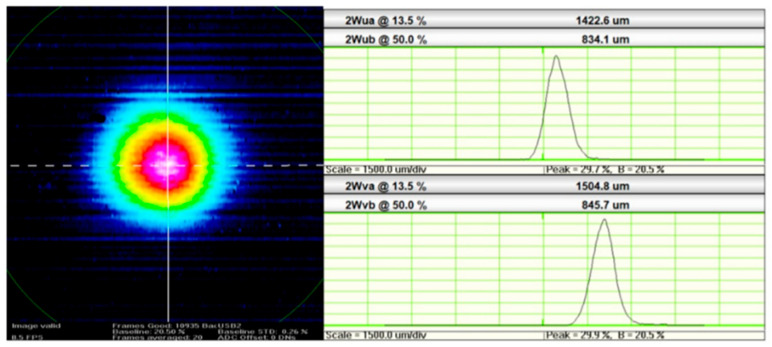
Image of a collimated output beam emitted by the constructed fiber laser.

The beam quality of the constructed laser was also investigated. The beam profile was measured using a silicon microbolometer camera (WinCamD™-FIR2-16-HR, Redding, CA, USA) operating in spectral region stretching between 2 and 16 µm. A collimated beam of around a 1.5 mm diameter illuminated the silicon microbolometer camera. The measured image of the collimated laser beam is presented in Figure 9. The measured beam profile has approximately a Gaussian distribution, which confirms that the constructed laser operates in a single-transverse mode.

In order to compare our results with the current state-of-the-art on actively Q-switched and gain-switched 3 µm fiber lasers, some recent achievements are summarized in Table 1. From Table 1, it can be concluded that our result is the best in terms of peak power obtained for a single-transverse mode 3 µm laser operating in an active Q-switching or gain-switching regime.

## 4. Impact

The generation of a high-energy nanosecond pulse in the mid-infrared region from a fiber laser source creates new application opportunities. Recently, [31] predicted that laser generating around 50 µJ output energy and pulse durations below 100 ns should be suitable for developing differential absorption lidar (DIAL). Pulsed mid-infrared fiber lasers have also the potential to be used in laser surgery. First, the developed laser reported in this contribution operates at ~2.8 µm, which is close to the peak of the water OH molecule vibration stretching mode. This ensures a low penetration depth. Furthermore, such a laser generates pulses with durations less than 100 ns, which is beneficial for laser surgery, because the heat diffusion during the laser pulse application is negligible. Moreover, in most publications devoted to laser surgery, the ablation rate is presented as a function of fluence. The calculated fluence for the laser developed in this contribution is ~28 J/cm^2^ (assuming output energy 50 µJ and core diameter of 15 µm). According to [32], the fluence in the range of 5 J/cm^2^ should be sufficient for cutaneous laser resurfacing, and the fluence in the range of 15 J/cm^2^ should trigger ablation in pig corneas [33]. The laser reported in this contribution is suitable for both applications.

## 5. Conclusions

In summary, actively Q-switched, single-transverse mode Er^3+^:ZBLAN fiber lasers operating at 2.78 µm were practically realized. The realized laser performance was investigated experimentally, and the dependence of laser characteristics on the pump power, fiber length, and repetition rate was discussed. When the gain fiber length is 1.1 m, the developed laser generates pulses with 0.821 kW peak power and 46 µJ pulse energy, with a pulse duration as short as 56 ns at the repetition rate of 10 kHz. To the best of the authors’ knowledge, a 0.821 kW peak power is the highest peak power achieved so far for actively Q-switched, single-transverse mode fiber lasers operating near a 3 µm wavelength. Future works will be devoted to scaling of the laser output power.

## Figures and Tables

**Table 1:** Progress of actively Q-switched and gain-switched 3 µm fiber lasers (AOM: acousto-optic modulator; GS: gain-switched; MM: multi-transverse mode; SM: single-transverse mode; and ZBLAN (composed of fluorides: ZrF_4_, BaF_2_, LaF_3_, AlF_3_, and NaF)).

Gain Medium	Modulation Device	Peak Power (kW)	Pulse Duration (ns)	Average Power (W)	Repetition Rate (kHz)	Year and Reference
Er^3+^:ZBLAN (35 µm core MM)	AOM	0.9	90	12	120	2011 [12]
Er^3+^:ZBLAN (33 µm core MM)	AOM	10.6	53	0.56	1	2015 [16]
Er^3+^:ZBLAN (15 µm core SM)	GS	0.42	170	11.2	140	2018 [13]
Ho^3+^/Pr^3+^:ZBLAN (10 µm core SM)	AOM	0.576	33	0.019	1	2013 [25]
Er^3+^:ZBLAN (15 µm core SM)	AOM	0.821	56	0.46	10	This work
